# 7-(4-Fluoro­benzyl­amino)-2-phenyl-1,2,4-triazolo[1,5-*a*][1,3,5]triazin-5-amine methanol disolvate^1^

**DOI:** 10.1107/S1600536811014176

**Published:** 2011-04-22

**Authors:** Anton V. Dolzhenko, Geok Kheng Tan, Lip Lin Koh, Anna V. Dolzhenko, Wai Keung Chui

**Affiliations:** a School of Pharmacy, Faculty of Health Sciences, Curtin University of Technology, GPO Box U1987, Perth 6845, Western Australia, Australia; b Department of Chemistry, Faculty of Science, National University of Singapore, 3 Science Drive 3, Singapore 117543, Singapore; c Perm State Pharmaceutical Academy, 2 Polevaya Street, Perm 614990, Russian Federation; d Department of Pharmacy, Faculty of Science, National University of Singapore, 18 Science Drive 4, Singapore 117543, Singapore

## Abstract

The 1,2,4-triazolo[1,5-*a*][1,3,5]triazine system in the title compound, C_17_H_14_FN_7_·2CH_3_OH, is essentially planar, with an r.m.s. deviation of 0.0215 Å. The attached phenyl ring lies almost in the mean plane of the heterocyclic core [dihedral angle = 3.56 (4)°]. In the crystal, centrosymmetric inversion dimers connected *via* inter­molecular N—H⋯N hydrogen bonds between H atom of the primary amino group and the triazine N atom [*R*_2_^2^(8) graph-set motif] form sheets parallel to (010). A second set of dimers connected *via* N—H⋯F hydrogen bonds between the other H atom of the primary amino group and the F atom forms an *R*_2_^2^(24) graph-set motif linking the sheets. Methanol solvent mol­ecules are packed in channels running along the [010] direction.

## Related literature

For a review of the synthesis and biological activity of 1,2,4-triazolo[1,5-*a*][1,3,5]triazines, see: Dolzhenko *et al.* (2006 ▶). For our work on the synthesis and biological activity of 1,2,4-triazolo[1,5-*a*][1,3,5]triazines, see: Dolzhenko *et al.* (2007*a* ▶,*b* ▶, 2008*a* ▶,*b* ▶, 2011*a* ▶). For the crystal structures of similar 1,2,4-triazolo[1,5-*a*][1,3,5]triazines, see: Dolzhenko *et al.* (2007*c* ▶,*d* ▶, 2008*c* ▶, 2011*b* ▶); Gilardi (1973 ▶); Khankischpur *et al.* (2010 ▶). For a review on the graph-set analysis of hydrogen bonding, see: Bernstein *et al.* (1995 ▶).
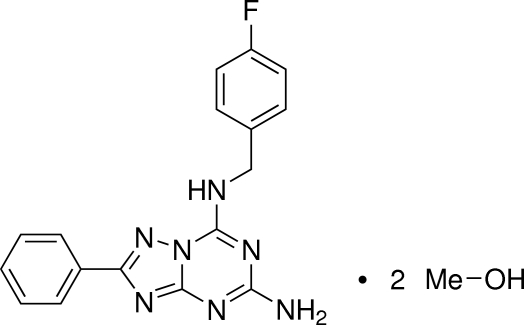


## Experimental

### 

#### Crystal data

C_17_H_14_FN_7_·2CH_4_O
*M* *_r_* = 399.44
Monoclinic, 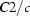

*a* = 27.516 (3) Å
*b* = 7.0091 (8) Å
*c* = 20.778 (3) Å
β = 104.380 (3)°
*V* = 3881.7 (8) Å^3^
*Z* = 8
Mo *K*α radiation
μ = 0.10 mm^−1^
*T* = 100 K
0.56 × 0.18 × 0.02 mm

#### Data collection

Bruker SMART APEX CCD diffractometer
Absorption correction: multi-scan (*SADABS*; Sheldrick, 2001 ▶) *T*_min_ = 0.946, *T*_max_ = 0.998
11861 measured reflections
3820 independent reflections
2876 reflections with *I* > 2σ(*I*)
*R*_int_ = 0.066

#### Refinement

*R*[*F* ^2^ > 2σ(*F* ^2^)] = 0.084
*wR*(*F* ^2^) = 0.183
*S* = 1.20
3820 reflections
275 parameters
3 restraints
H atoms treated by a mixture of independent and constrained refinement
Δρ_max_ = 0.41 e Å^−3^
Δρ_min_ = −0.27 e Å^−3^

### 

Data collection: *SMART* (Bruker, 2001 ▶); cell refinement: *SAINT* (Bruker, 2001 ▶); data reduction: *SAINT*; program(s) used to solve structure: *SHELXS97* (Sheldrick, 2008 ▶); program(s) used to refine structure: *SHELXL97* (Sheldrick, 2008 ▶); molecular graphics: *SHELXTL* (Sheldrick, 2008 ▶); software used to prepare material for publication: *SHELXTL*.

## Supplementary Material

Crystal structure: contains datablocks I, global. DOI: 10.1107/S1600536811014176/sj5125sup1.cif

Structure factors: contains datablocks I. DOI: 10.1107/S1600536811014176/sj5125Isup2.hkl

Supplementary material file. DOI: 10.1107/S1600536811014176/sj5125Isup3.cdx

Additional supplementary materials:  crystallographic information; 3D view; checkCIF report

## Figures and Tables

**Table 1:** Hydrogen-bond geometry (Å, °)

*D*—H⋯*A*	*D*—H	H⋯*A*	*D*⋯*A*	*D*—H⋯*A*
N6—H6*B*⋯O2*S*^i^	0.87 (2)	2.52 (3)	3.033 (4)	118 (3)
N6—H6*B*⋯F1^ii^	0.87 (2)	2.48 (2)	3.307 (3)	159 (3)
N6—H6*A*⋯N5^i^	0.90 (2)	2.13 (2)	3.025 (4)	178 (3)
N7—H7N⋯O1*S*^iii^	0.88 (2)	1.96 (2)	2.797 (4)	158 (3)
O1*S*—H1*S*⋯O2*S*	0.84	1.86	2.690 (3)	169
O2*S*—H2*S*⋯N2	0.84	1.91	2.731 (4)	166

Symmetry codes: (i) 
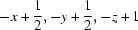
; (ii) 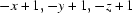; (iii) 
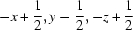
.

**Fig. 1.**
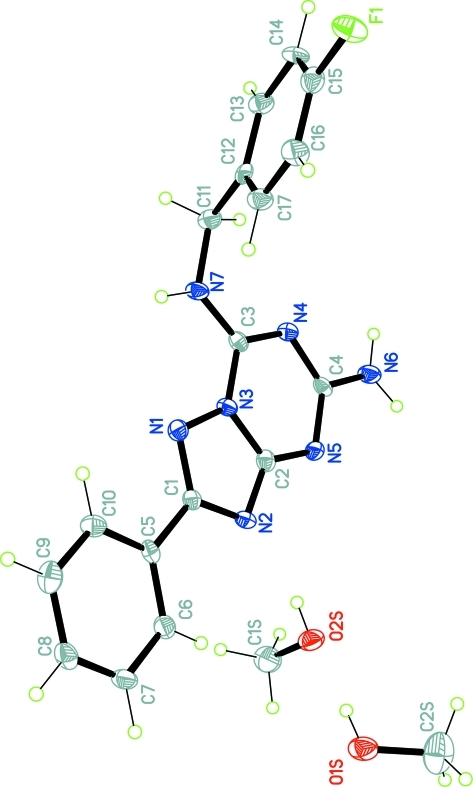
The molecular structure of 7-(4-fluorobenzylamino)-2-phenyl-1,2,4-triazolo[1,5-a][1,3,5]triazine-5-amine methanol disolvate showing the atomic numbering scheme. Displacement ellipsoids are drawn at the 50% probability level.

**Fig. 2.**
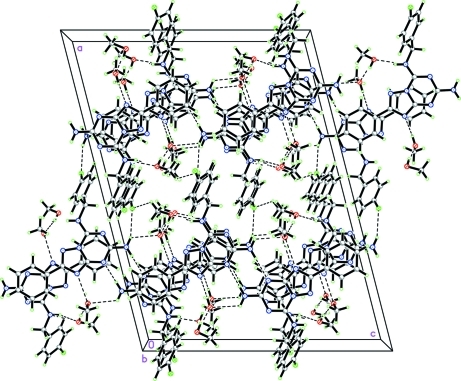
Crystal packing in the cell (view along axis b).

## supplementary crystallographic information

### Comment

The 1,2,4-triazolo[1,5-*a*]triazine heterocyclic system has been well recognized as a promising scaffold for the construction of compounds with diverse biological effects (Dolzhenko *et al.*, 2006). In our search for potential therapeutic agents in this class of compounds we devised a number of effective methods for the preparation of 1,2,4-triazolo[1,5-*a*]triazines (Dolzhenko *et al.*, 2007*a*,*b*; Dolzhenko *et al.*, 2008*a*,*b*). The structural investigations of 1,2,4-triazolo[1,5-*a*]triazines include an earlier report (Gilardi, 1973) of the 5,7-bis(dimethylamino)-2-methylthio-1,2,4-triazolo[1,5-*a*]triazine structure, our publications regarding structures of various amino substituted 1,2,4-triazolo[1,5-*a*]triazines (Dolzhenko *et al.*, 2007*c*,*d*; Dolzhenko *et al.*, 2008*c*; Dolzhenko *et al.*, 2011*b*), and a recent paper (Khankischpur *et al.*, 2010) mentioning the 2-amino-5-(2-phenylethyl)[1,2,4]triazolo[1,5-*a*] [1,3,5]triazin-7(6*H*)-one structure. In continuation of our program on the synthesis and structural investigation of potentially bioactive 1,2,4-triazolo[1,5-*a*]triazines, we synthesized 7-(4-fluorobenzylamino)-2-phenyl-1,2,4-triazolo[1,5-*a*][1,3,5]triazine-5-amine using a recently developed method (Dolzhenko *et al.*, 2008*a*) and report herein its molecular and crystal structure.

7-(4-Fluorobenzylamino)-2-phenyl-1,2,4-triazolo[1,5-*a*][1,3,5]triazine-5-amine crystallizes together with two methanol molecules (Fig. 1 & 2). The closely similar 7-dimethylamino-1,2,4-triazolo[1,5-*a*][1,3,5]triazin-5-amine reported earlier (Dolzhenko *et al.*, 2008*c*) also crystallized in the form of a methanol solvate. The 1,2,4-triazolo[1,5-*a*][1,3,5]triazine heterocyclic system is essentially planar with an r.m.s. deviation of 0.0215 Å. The phenyl ring mean plane C5—C10 makes a small dihedral angle of 3.56 (4)° with the mean plane of the 1,2,4-triazolo[1,5-*a*][1,3,5]triazine system. The amino group nitrogen atoms N6 and N7 are located practically in the plane of the heterocyclic core with slight deviations of 0.0861 (41) Å above and 0.0663 (41) Å below the mean plane, correspondingly. The molecule is twisted at the aminomethyl bridge N7—C11 [C3—N7—C11—C12 torsion angle is 100.38 (36)°].

In the crystal, molecules of 7-(4-fluorobenzylamino)-2-phenyl-1,2,4-triazolo[1,5-*a*][1,3,5]triazine-5-amine form two types of centrosymmetric inversion dimers (Fig. 2). The triazine N5 atom is connected with amino group N6—H6A of a neighbouring molecule by intermolecular N–H···N hydrogen bond making *R*^2^_2_(8) graph-set motif (Bernstein *et al.*, 1995) arranging the molecules in sheets parallel to the (010) plane. A second set of dimers connected *via* N–H···F hydrogen bonding between the N6—H6A amino group and the F1 atom of an adjacent molecule forms a *R*^2^_2_(24) graph-set motif linking the sheets. The methanol molecules are packed in channels running along the [010] direction and also participate in linking the sheets *via* O–H···N and N–H···O contacts.

### Experimental

The title compound was prepared according to the previously reported general method (Dolzhenko *et al.*, 2008*a*). 2-Phenyl-7-trichloromethyl-1,2,4-triazolo[1,5-*a*][1,3,5]triazin-5-amine (0.66 g, 2.0 mmol) was added to a solution of 4-fluorobenzylamine (0.28 ml, 2.5 mmol) in DMF (5 ml) and the mixture was heated at 70–80 °C with stirring for 3 h. After cooling, ice-cold water (40 ml) was added and the product was filtered and recrystallized from methanol.

### Refinement

All C-bound H atoms were positioned geometrically and included in the refinement using the riding-motion approximation [0.95 Å for CH of aromatic rings, 0.99 Å for methylene protons, 0.98 Å for methyl groups, and 0.84 Å for hydroxyl groups; *U*_iso_(H) = 1.2*U*_eq_(C_Ar_, *C*_methylenic_) and *U*_iso_(H) = 1.5*U*_eq_(O,*C*_Me_)] while the amino group H atoms were located in a difference map and refined with restraints on the bond lengths and thermal parameters.

### Crystal data

**Table:**

C_17_H_14_FN_7_·2CH_4_O	*F*(000) = 1680
*M**_r_* = 399.44	*D*_x_ = 1.367 Mg m^−^^3^
Monoclinic, *C*2/*c*	Melting point: 523 K
Hall symbol: -C 2yc	Mo *K*α radiation, λ = 0.71073 Å
*a* = 27.516 (3) Å	Cell parameters from 653 reflections
*b* = 7.0091 (8) Å	θ = 2.8–23.5°
*c* = 20.778 (3) Å	µ = 0.10 mm^−^^1^
β = 104.380 (3)°	*T* = 100 K
*V* = 3881.7 (8) Å^3^	Thin plate, colourless
*Z* = 8	0.56 × 0.18 × 0.02 mm

### Data collection

**Table:**

Bruker SMART APEX CCD diffractometer	3820 independent reflections
Radiation source: fine-focus sealed tube	2876 reflections with *I* > 2σ(*I*)
graphite	*R*_int_ = 0.066
φ and ω scans	θ_max_ = 26.0°, θ_min_ = 2.0°
Absorption correction: multi-scan (*SADABS*; Sheldrick, 2001)	*h* = −31→33
*T*_min_ = 0.946, *T*_max_ = 0.998	*k* = −8→8
11861 measured reflections	*l* = −25→25

### Refinement

**Table:**

Refinement on *F*^2^	Primary atom site location: structure-invariant direct methods
Least-squares matrix: full	Secondary atom site location: difference Fourier map
*R*[*F*^2^ > 2σ(*F*^2^)] = 0.084	Hydrogen site location: inferred from neighbouring sites
*wR*(*F*^2^) = 0.183	H atoms treated by a mixture of independent and constrained refinement
*S* = 1.20	*w* = 1/[σ^2^(*F*_o_^2^) + (0.067*P*)^2^ + 5.3197*P*] where *P* = (*F*_o_^2^ + 2*F*_c_^2^)/3
3820 reflections	(Δ/σ)_max_ < 0.001
275 parameters	Δρ_max_ = 0.41 e Å^−^^3^
3 restraints	Δρ_min_ = −0.27 e Å^−^^3^

### Special details

**Table:**

Geometry. All e.s.d.'s (except the e.s.d. in the dihedral angle between two l.s. planes) are estimated using the full covariance matrix. The cell e.s.d.'s are taken into account individually in the estimation of e.s.d.'s in distances, angles and torsion angles; correlations between e.s.d.'s in cell parameters are only used when they are defined by crystal symmetry. An approximate (isotropic) treatment of cell e.s.d.'s is used for estimating e.s.d.'s involving l.s. planes.
Refinement. Refinement of *F*^2^ against ALL reflections. The weighted *R*-factor *wR* and goodness of fit *S* are based on *F*^2^, conventional *R*-factors *R* are based on *F*, with *F* set to zero for negative *F*^2^. The threshold expression of *F*^2^ > σ(*F*^2^) is used only for calculating *R*-factors(gt) *etc*. and is not relevant to the choice of reflections for refinement. *R*-factors based on *F*^2^ are statistically about twice as large as those based on *F*, and *R*- factors based on ALL data will be even larger.

### Fractional atomic coordinates and isotropic or equivalent isotropic displacement parameters (Å^2^)

**Table:**

	*x*	*y*	*z*	*U*_iso_*/*U*_eq_	
F1	0.55659 (7)	0.8048 (3)	0.40137 (10)	0.0281 (5)	
N1	0.31099 (10)	0.1280 (4)	0.27731 (12)	0.0158 (6)	
N2	0.23799 (10)	0.1664 (4)	0.30860 (13)	0.0154 (6)	
N3	0.31934 (10)	0.1366 (4)	0.34551 (12)	0.0147 (6)	
N4	0.36437 (10)	0.1522 (4)	0.45512 (13)	0.0167 (6)	
N5	0.27356 (10)	0.1770 (4)	0.42739 (13)	0.0163 (6)	
N6	0.32075 (11)	0.1989 (4)	0.53427 (14)	0.0211 (7)	
H6A	0.2925 (10)	0.233 (5)	0.5459 (17)	0.025*	
H6B	0.3506 (9)	0.196 (5)	0.5615 (15)	0.025*	
N7	0.40528 (10)	0.1013 (4)	0.37144 (13)	0.0176 (6)	
H7N	0.4053 (13)	0.084 (5)	0.3297 (10)	0.021*	
C1	0.26169 (12)	0.1470 (4)	0.25817 (15)	0.0139 (7)	
C2	0.27519 (12)	0.1601 (5)	0.36343 (16)	0.0162 (7)	
C3	0.36411 (12)	0.1303 (5)	0.39200 (15)	0.0150 (7)	
C4	0.31899 (12)	0.1752 (4)	0.46998 (15)	0.0151 (7)	
C5	0.23498 (12)	0.1490 (5)	0.18730 (15)	0.0150 (7)	
C6	0.18294 (12)	0.1645 (5)	0.16728 (16)	0.0187 (7)	
H6	0.1641	0.1717	0.1998	0.022*	
C7	0.15854 (13)	0.1694 (5)	0.10072 (16)	0.0209 (8)	
H7	0.1230	0.1805	0.0876	0.025*	
C8	0.18578 (13)	0.1582 (5)	0.05306 (16)	0.0205 (8)	
H8	0.1690	0.1636	0.0072	0.025*	
C9	0.23750 (14)	0.1392 (5)	0.07209 (16)	0.0225 (8)	
H9	0.2561	0.1293	0.0394	0.027*	
C10	0.26205 (13)	0.1346 (5)	0.13909 (16)	0.0189 (7)	
H10	0.2975	0.1216	0.1521	0.023*	
C11	0.45495 (12)	0.1136 (5)	0.41539 (16)	0.0192 (8)	
H11A	0.4756	0.0075	0.4052	0.023*	
H11B	0.4524	0.0969	0.4617	0.023*	
C12	0.48156 (12)	0.3013 (5)	0.41021 (15)	0.0161 (7)	
C13	0.52866 (12)	0.3337 (5)	0.45154 (16)	0.0196 (8)	
H13	0.5439	0.2371	0.4820	0.024*	
C14	0.55430 (12)	0.5031 (5)	0.44964 (16)	0.0195 (8)	
H14	0.5864	0.5251	0.4787	0.023*	
C15	0.53142 (13)	0.6384 (5)	0.40394 (17)	0.0209 (8)	
C16	0.48547 (13)	0.6132 (5)	0.36150 (16)	0.0223 (8)	
H16	0.4711	0.7092	0.3303	0.027*	
C17	0.46011 (13)	0.4430 (5)	0.36504 (16)	0.0200 (8)	
H17	0.4278	0.4233	0.3363	0.024*	
O1S	0.07528 (9)	0.4762 (4)	0.24754 (11)	0.0271 (6)	
H1S	0.0990	0.4061	0.2677	0.041*	
C1S	0.12533 (14)	0.0350 (6)	0.31845 (18)	0.0310 (9)	
H1S1	0.1364	−0.0167	0.3635	0.047*	
H1S2	0.1395	−0.0416	0.2881	0.047*	
H1S3	0.0886	0.0311	0.3041	0.047*	
O2S	0.14190 (9)	0.2264 (4)	0.31791 (12)	0.0250 (6)	
H2S	0.1720	0.2276	0.3160	0.037*	
C2S	0.06242 (18)	0.6058 (6)	0.2930 (2)	0.0419 (11)	
H2S1	0.0920	0.6315	0.3293	0.063*	
H2S2	0.0359	0.5501	0.3111	0.063*	
H2S3	0.0504	0.7253	0.2701	0.063*	

### Atomic displacement parameters (Å^2^)

**Table:**

	*U*^11^	*U*^22^	*U*^33^	*U*^12^	*U*^13^	*U*^23^
F1	0.0251 (12)	0.0258 (12)	0.0301 (11)	−0.0071 (9)	0.0004 (9)	0.0057 (10)
N1	0.0165 (15)	0.0191 (15)	0.0120 (13)	0.0012 (12)	0.0037 (11)	−0.0003 (12)
N2	0.0127 (14)	0.0177 (15)	0.0139 (13)	−0.0010 (11)	−0.0002 (11)	−0.0008 (12)
N3	0.0129 (14)	0.0170 (15)	0.0131 (13)	−0.0014 (11)	0.0011 (11)	−0.0006 (11)
N4	0.0138 (14)	0.0184 (15)	0.0163 (14)	0.0000 (11)	0.0010 (11)	−0.0006 (12)
N5	0.0143 (14)	0.0204 (16)	0.0148 (13)	0.0011 (11)	0.0048 (11)	−0.0021 (12)
N6	0.0152 (16)	0.0319 (18)	0.0147 (15)	−0.0005 (13)	0.0007 (12)	−0.0026 (13)
N7	0.0131 (14)	0.0277 (17)	0.0109 (13)	−0.0019 (12)	0.0008 (11)	−0.0006 (12)
C1	0.0154 (17)	0.0081 (16)	0.0177 (16)	−0.0018 (12)	0.0031 (13)	−0.0010 (13)
C2	0.0189 (17)	0.0117 (17)	0.0184 (17)	−0.0014 (13)	0.0052 (14)	−0.0007 (14)
C3	0.0170 (17)	0.0117 (16)	0.0158 (16)	−0.0037 (13)	0.0034 (13)	−0.0009 (13)
C4	0.0173 (17)	0.0123 (17)	0.0127 (15)	−0.0007 (13)	−0.0017 (13)	0.0002 (13)
C5	0.0202 (18)	0.0114 (16)	0.0125 (16)	−0.0012 (13)	0.0024 (13)	−0.0015 (13)
C6	0.0196 (18)	0.0182 (19)	0.0183 (17)	−0.0015 (14)	0.0048 (14)	−0.0030 (14)
C7	0.0156 (18)	0.0212 (19)	0.0219 (18)	0.0011 (14)	−0.0030 (14)	−0.0017 (15)
C8	0.028 (2)	0.0187 (19)	0.0119 (16)	0.0035 (15)	−0.0005 (14)	0.0009 (14)
C9	0.032 (2)	0.022 (2)	0.0171 (17)	0.0009 (16)	0.0130 (15)	0.0038 (15)
C10	0.0168 (17)	0.0211 (19)	0.0170 (17)	0.0009 (14)	0.0011 (14)	0.0031 (15)
C11	0.0147 (17)	0.0240 (19)	0.0183 (17)	0.0013 (14)	0.0032 (14)	0.0014 (15)
C12	0.0156 (17)	0.0228 (19)	0.0129 (15)	0.0015 (14)	0.0092 (13)	−0.0034 (14)
C13	0.0158 (17)	0.027 (2)	0.0157 (16)	0.0011 (14)	0.0030 (13)	0.0026 (15)
C14	0.0111 (17)	0.026 (2)	0.0186 (17)	−0.0029 (14)	−0.0023 (13)	0.0000 (15)
C15	0.0234 (19)	0.0201 (19)	0.0214 (17)	−0.0002 (15)	0.0099 (15)	−0.0005 (15)
C16	0.0226 (19)	0.024 (2)	0.0169 (17)	0.0037 (15)	−0.0018 (14)	0.0031 (15)
C17	0.0159 (17)	0.027 (2)	0.0162 (16)	−0.0003 (15)	0.0017 (14)	0.0002 (15)
O1S	0.0222 (14)	0.0407 (17)	0.0166 (12)	0.0037 (12)	0.0014 (10)	0.0019 (12)
C1S	0.026 (2)	0.044 (3)	0.025 (2)	−0.0040 (18)	0.0104 (17)	−0.0041 (18)
O2S	0.0158 (13)	0.0364 (16)	0.0235 (13)	0.0028 (11)	0.0062 (11)	−0.0015 (12)
C2S	0.065 (3)	0.027 (2)	0.038 (2)	0.006 (2)	0.020 (2)	0.0071 (19)

### Geometric parameters (Å, °)

**Table:**

F1—C15	1.364 (4)	C9—C10	1.389 (5)
N1—C1	1.322 (4)	C9—H9	0.9500
N1—N3	1.379 (3)	C10—H10	0.9500
N2—C2	1.330 (4)	C11—C12	1.523 (5)
N2—C1	1.371 (4)	C11—H11A	0.9900
N3—C3	1.364 (4)	C11—H11B	0.9900
N3—C2	1.366 (4)	C12—C13	1.384 (5)
N4—C3	1.319 (4)	C12—C17	1.393 (5)
N4—C4	1.368 (4)	C13—C14	1.387 (5)
N5—C4	1.340 (4)	C13—H13	0.9500
N5—C2	1.346 (4)	C14—C15	1.378 (5)
N6—C4	1.335 (4)	C14—H14	0.9500
N6—H6A	0.900 (18)	C15—C16	1.362 (5)
N6—H6B	0.874 (18)	C16—C17	1.393 (5)
N7—C3	1.322 (4)	C16—H16	0.9500
N7—C11	1.445 (4)	C17—H17	0.9500
N7—H7N	0.877 (18)	O1S—C2S	1.418 (5)
C1—C5	1.473 (4)	O1S—H1S	0.8400
C5—C6	1.392 (5)	C1S—O2S	1.418 (5)
C5—C10	1.393 (4)	C1S—H1S1	0.9800
C6—C7	1.380 (5)	C1S—H1S2	0.9800
C6—H6	0.9500	C1S—H1S3	0.9800
C7—C8	1.385 (5)	O2S—H2S	0.8400
C7—H7	0.9500	C2S—H2S1	0.9800
C8—C9	1.386 (5)	C2S—H2S2	0.9800
C8—H8	0.9500	C2S—H2S3	0.9800
			
C1—N1—N3	101.6 (2)	C9—C10—H10	119.8
C2—N2—C1	103.9 (3)	C5—C10—H10	119.8
C3—N3—C2	121.2 (3)	N7—C11—C12	113.7 (3)
C3—N3—N1	128.1 (3)	N7—C11—H11A	108.8
C2—N3—N1	110.7 (2)	C12—C11—H11A	108.8
C3—N4—C4	117.3 (3)	N7—C11—H11B	108.8
C4—N5—C2	113.4 (3)	C12—C11—H11B	108.8
C4—N6—H6A	119 (2)	H11A—C11—H11B	107.7
C4—N6—H6B	116 (2)	C13—C12—C17	118.4 (3)
H6A—N6—H6B	124 (3)	C13—C12—C11	119.4 (3)
C3—N7—C11	122.6 (3)	C17—C12—C11	122.2 (3)
C3—N7—H7N	124 (2)	C12—C13—C14	121.8 (3)
C11—N7—H7N	114 (2)	C12—C13—H13	119.1
N1—C1—N2	115.3 (3)	C14—C13—H13	119.1
N1—C1—C5	121.4 (3)	C15—C14—C13	117.4 (3)
N2—C1—C5	123.3 (3)	C15—C14—H14	121.3
N2—C2—N5	129.5 (3)	C13—C14—H14	121.3
N2—C2—N3	108.5 (3)	C16—C15—F1	119.0 (3)
N5—C2—N3	122.0 (3)	C16—C15—C14	123.3 (3)
N4—C3—N7	123.1 (3)	F1—C15—C14	117.7 (3)
N4—C3—N3	118.8 (3)	C15—C16—C17	118.3 (3)
N7—C3—N3	118.1 (3)	C15—C16—H16	120.9
N6—C4—N5	117.1 (3)	C17—C16—H16	120.9
N6—C4—N4	115.6 (3)	C12—C17—C16	120.8 (3)
N5—C4—N4	127.3 (3)	C12—C17—H17	119.6
C6—C5—C10	119.0 (3)	C16—C17—H17	119.6
C6—C5—C1	121.3 (3)	C2S—O1S—H1S	109.5
C10—C5—C1	119.7 (3)	O2S—C1S—H1S1	109.5
C7—C6—C5	120.7 (3)	O2S—C1S—H1S2	109.5
C7—C6—H6	119.7	H1S1—C1S—H1S2	109.5
C5—C6—H6	119.7	O2S—C1S—H1S3	109.5
C6—C7—C8	120.0 (3)	H1S1—C1S—H1S3	109.5
C6—C7—H7	120.0	H1S2—C1S—H1S3	109.5
C8—C7—H7	120.0	C1S—O2S—H2S	109.5
C7—C8—C9	120.1 (3)	O1S—C2S—H2S1	109.5
C7—C8—H8	120.0	O1S—C2S—H2S2	109.5
C9—C8—H8	120.0	H2S1—C2S—H2S2	109.5
C8—C9—C10	119.8 (3)	O1S—C2S—H2S3	109.5
C8—C9—H9	120.1	H2S1—C2S—H2S3	109.5
C10—C9—H9	120.1	H2S2—C2S—H2S3	109.5
C9—C10—C5	120.4 (3)		
			
C1—N1—N3—C3	−177.9 (3)	N1—C1—C5—C6	178.5 (3)
C1—N1—N3—C2	0.0 (3)	N2—C1—C5—C6	−2.3 (5)
N3—N1—C1—N2	−0.2 (4)	N1—C1—C5—C10	−1.4 (5)
N3—N1—C1—C5	179.1 (3)	N2—C1—C5—C10	177.9 (3)
C2—N2—C1—N1	0.4 (4)	C10—C5—C6—C7	−1.3 (5)
C2—N2—C1—C5	−179.0 (3)	C1—C5—C6—C7	178.9 (3)
C1—N2—C2—N5	178.2 (3)	C5—C6—C7—C8	0.2 (5)
C1—N2—C2—N3	−0.3 (3)	C6—C7—C8—C9	1.0 (5)
C4—N5—C2—N2	−176.2 (3)	C7—C8—C9—C10	−1.1 (5)
C4—N5—C2—N3	2.2 (4)	C8—C9—C10—C5	0.0 (5)
C3—N3—C2—N2	178.3 (3)	C6—C5—C10—C9	1.2 (5)
N1—N3—C2—N2	0.2 (4)	C1—C5—C10—C9	−179.0 (3)
C3—N3—C2—N5	−0.4 (5)	C3—N7—C11—C12	−100.4 (4)
N1—N3—C2—N5	−178.4 (3)	N7—C11—C12—C13	177.6 (3)
C4—N4—C3—N7	−178.0 (3)	N7—C11—C12—C17	−2.4 (4)
C4—N4—C3—N3	1.6 (4)	C17—C12—C13—C14	1.0 (5)
C11—N7—C3—N4	−7.6 (5)	C11—C12—C13—C14	−179.0 (3)
C11—N7—C3—N3	172.8 (3)	C12—C13—C14—C15	−1.1 (5)
C2—N3—C3—N4	−1.7 (5)	C13—C14—C15—C16	0.2 (5)
N1—N3—C3—N4	176.0 (3)	C13—C14—C15—F1	−179.4 (3)
C2—N3—C3—N7	177.9 (3)	F1—C15—C16—C17	−179.6 (3)
N1—N3—C3—N7	−4.4 (5)	C14—C15—C16—C17	0.8 (5)
C2—N5—C4—N6	177.4 (3)	C13—C12—C17—C16	0.0 (5)
C2—N5—C4—N4	−2.4 (5)	C11—C12—C17—C16	−179.9 (3)
C3—N4—C4—N6	−179.3 (3)	C15—C16—C17—C12	−0.9 (5)
C3—N4—C4—N5	0.5 (5)		

### Hydrogen-bond geometry (Å, °)

**Table:**

*D*—H···*A*	*D*—H	H···*A*	*D*···*A*	*D*—H···*A*
N6—H6B···O2S^i^	0.87 (2)	2.52 (3)	3.033 (4)	118 (3)
N6—H6B···F1^ii^	0.87 (2)	2.48 (2)	3.307 (3)	159 (3)
N6—H6A···N5^i^	0.90 (2)	2.13 (2)	3.025 (4)	178 (3)
N7—H7N···N1	0.88 (2)	2.57 (3)	2.841 (4)	99 (2)
N7—H7N···O1S^iii^	0.88 (2)	1.96 (2)	2.797 (4)	158 (3)
O1S—H1S···O2S	0.84	1.86	2.690 (3)	169
O2S—H2S···N2	0.84	1.91	2.731 (4)	166
N7—H7N···N1	0.88 (2)	2.57 (3)	2.841 (4)	99 (2)

Symmetry codes: (i) −*x*+1/2, −*y*+1/2, −*z*+1; (ii) −*x*+1, −*y*+1, −*z*+1; (iii) −*x*+1/2, *y*−1/2, −*z*+1/2.
